# Undesired Births, Contraception, and Abortion Before and After the Cairo Consensus: Trends in Conditional Undesired Birth Rates and the Impact of Contraception and Abortion

**DOI:** 10.1111/sifp.70014

**Published:** 2025-05-20

**Authors:** Jonathan Marc Bearak, Ellie Leong, Jewel Gausman, Jessica Rosenberg, Mariah Menanno, Samira Sackietey, Octavia Mulhern, Vladimíra Kantorová, Joseph Molitoris

## Abstract

The Programme of Action adopted after the International Conference on Population and Development (ICPD), and later the Beijing Declaration, affirmed commitments to the human right to decide on the number and spacing of one's children and have the information and means to do so. In this study, we estimate trends related to this component of reproductive agency—undesired births per thousand women who want to avoid pregnancy, the conditional undesired birth rate—with annual rates for five‐year periods from 1975 to 2024. Worldwide, 36 million undesired births occurred annually in 2020–2024 compared to 45 million annually in 1990–1994, corresponding to a decrease in rate from 61 to 32. Had it not been for increases in contraceptive use since 1990–1994, the global average rate in 2020–2024 would have been 36 percent higher than it actually was. Had it not been for increasing proportions of pregnancies aborted, the rate would have been 58 percent higher. Comparing regional averages, excepting Sub‐Saharan Africa and Oceania, the pace of decline in conditional undesired birth rates slowed by the 2000s; hence, the global average rate decreased by 22 percent in the latter half of the post‐ICPD period after declining by 31 percent and 33 percent during the 15‐year periods immediately before and after ICPD.

## INTRODUCTION

Adopting the Programme of Action at the International Conference on Population and Development (ICPD) in Cairo in 1994 by 179 governments marked a shift towards the centrality of human rights in population and development policies and programs. These include the reproductive right of all individuals and couples to decide on the number and spacing of their children and have the information and means to do so (Principle 8 of the Programme of Action, United Nations 1995). The unanimous adoption of the Beijing Declaration by delegates from 189 countries at the Fourth World Conference on Women in 1995 reaffirmed this principle, declaring, “the right of all women to control all aspects of their health, in particular their own fertility, is basic to their empowerment” (United Nations 1996). Enabling and supporting this right requires that governments and the global health and development communities work to ensure access to comprehensive reproductive health services, including but not limited to contraception and safe abortion.

Several efforts to quantify global improvements in reproductive health followed within the monitoring frameworks of the Millennium Development Goals (United Nations 2007) and Sustainable Development Goals (SDG; United Nations 2015). Advances in data availability and statistical modeling coincided with increased commitment within the international community to the global monitoring of reproductive health. This led to the development of internationally comparable estimates of the proportions of reproductive‐age women using traditional or modern contraception and the proportions wanting to avoid pregnancy, published and regularly updated by the United Nations (Alkema et al. 2013; Cahill et al. 2017; Kantorová et al. 2020; United Nations Department of Economic and Social Affairs, Population Division 2024a). Subsequently, the Guttmacher Institute and World Health Organization (WHO) collaborated to produce a corresponding set of global estimates covering pregnancy by outcome and desire—whether a woman wanted to become pregnant in contrast to wanting to postpone, space, or stop childbearing (Bearak et al. 2019, 2020, 2022). These studies found substantial increases in contraceptive use and decreases in the incidence of undesired pregnancy. Other research found that maternal mortality decreased worldwide over the same period (WHO 2023). Taken together, the estimates produced by these studies suggest substantial improvements in women's reproductive health outcomes and realization of their reproductive desires.

Throughout this time, the decline in global fertility continued to unfold. Total fertility decreased to 2.25 live births per woman in 2024 from 4.08 live births per woman, on average, in 1975. Today, two‐thirds of the world's population lives in a country or area with total fertility below 2.1 live births per woman (United Nations Department of Economic and Social Affairs, Population Division 2024b). This contributes to increasing concerns about low fertility in some countries, contrasting with the ongoing concerns about population growth in others. Political and economic pressures contribute to changes in where funding sources target limited investments, while technological advancements in contraception and abortion, such as hormonal implants, vacuum aspiration, and medication abortion, increase options for regulating fertility (Anderson and Johnston 2023; Ganatra et al. 2017; Mayall and Fine 2014; Starrs et al. 2018; Stenberg et al. 2017; Wexler et al. 2024). Abortion policies regressed in four countries, the United States, El Salvador, Nicaragua, and Poland, contrasting with a broader global trend towards reform, with liberalization occurring in 30 countries throughout Asia, Western Europe, sub‐Saharan Africa, and Latin America, where the Green Wave led to liberalization in several countries (Center for Reproductive Rights 2023). Meanwhile, medication abortion contributed to landmark progress in the safety and availability of abortions in countries that prohibit abortion. How these factors affect trends in undesired births could vary considerably across regions.

This study examines the impact of contraception and abortion on undesired births over time. accounting for changes over time in reproductive desires. To do this, we analyze trends in the conditional undesired birth rate. This metric relates the number of undesired births to the number of women who want to avoid becoming pregnant. Specifically, it divides one thousand times the sum of these births by the number of other married or sexually active fecund women who reported wanting to postpone, space, or stop childbearing.

To produce estimates, we use the Bayesian hierarchical model developed by the Guttmacher Institute and WHO to estimate the number of pregnancies by desire and outcome (Bearak et al. 2019, 2020, 2022). We input newly available data into the model, as well as historical data from the 1970s and 1980s not previously used, to generate annual average estimates of the conditional undesired birth rate for five‐year periods from 1975 and 2024. This model estimates undesired births as a function of the United Nations‐estimated family planning indicators (United Nations Department of Economic and Social Affairs, Population Division 2024a) and the proportions of undesired pregnancies ending in abortion. To do this, the model produces period‐specific estimates of the relationship between family planning indicators and undesired pregnancy and of the odds of abortion conditional on pregnancy, fit to data on overall birth rates, the proportions of births from desired pregnancies, and abortion incidence.

With this model, we can calculate counterfactual undesired birth rates by applying parameter estimates from earlier periods to the present. Comparing counterfactuals for 2020–2024 to corresponding empirical estimates produces an estimate of the impact of contraception or abortion, depending on which parameters we let vary, by producing an estimate of how different the undesired birth rates would have been—higher, similar, or lower—had contraception or abortion not changed.

## ANTECEDENT LITERATURE ON CONDITIONAL PREGNANCY AND BIRTH RATES

A previous article introduced the corresponding *pregnancy* rate, the conditional undesired pregnancy rate (Bearak et al. 2023). They examined how the conventional focus on outcome prevalence among all women of reproductive age did not account for differences across countries nor over time in reproductive desires and vastly understated disparities across the world in the incidence of undesired pregnancy to women who wanted to avoid pregnancy, as well as the progress made after the 1990s in the regions with the highest rates.

In another analysis, Casterline and El‐Zeini (2022), analyzing fertility among women who wanted to *stop* childbearing in low‐ and middle‐income countries, developed a conditional *unwanted fertility* rate. Using this indicator, they analyzed trends in unwanted fertility from the 1970s through the 2010s and found decreases in Sub‐Saharan Africa that were similar to trends in Asia and Latin America. In contrast, using outcome prevalence among all women of reproductive age to perform the analysis would result in finding no change in unwanted fertility in sub‐Saharan Africa.

Our study's conditional undesired birth rate corresponds to the conditional undesired pregnancy rate from Bearak and colleagues. It contrasts with the conditional unwanted fertility rate analyzed by Casterline and El Zeini because we include births to women who wanted to postpone or space childbearing. We use this metric because of our interest in the right to regulate fertility and the substantial body of evidence on their associations with health and socioeconomic outcomes.

## MEASUREMENT LANGUAGE

Meaningful debates about how to characterize pregnancies exist in part as a response to the use of global indicators to measure progress towards the fulfillment of reproductive rights. Most literature describes undesired pregnancies as “unintended” and the corresponding births as “unplanned.” Limitations in using “unintended” and “unplanned” to characterize pregnancies have been identified in the literature that notes variability in how women interpret and define these terms. Some argue that using such terms may not adequately represent women's attitudes towards pregnancy nor be appropriately derived from the related survey questions (Barrett and Wellings 2002; Fischer et al. 1999; Klerman 2000; Santelli et al. 2003; Aiken et al. 2016; Mumford et al. 2016; Kost and Zolna 2019; Rackin and Morgan 2018; Dehlendorf et al. 2018; Bearak et al. 2021; Fabic 2022). Both the intentional use and careful interpretation of language when measuring and discussing pregnancy and birth are central to supporting women's reproductive health and rights. Hence, we employ the adjective *undesired*, and the phrase *wanted to avoid pregnancy*, in place of the established terms *unplanned* and *in need of family planning (or contraception)*, respectively, to more accurately reflect the questions posed to women in surveys. Accordingly, the terms used in this paper differ from those used usually for family planning indicators (see Table 1).

**TABLE 1:** Description of indicators terminology

Term used	Also known as	Description
Women who wanted to avoid pregnancy	Total demand or need for family planning	This indicator serves as the denominator for the Sustainable Development Goal indicator for contraceptive use (indicator 3.7.1) and the conditional undesired pregnancy and birth rates. Scholars proposed this phrasing in place of established phrasing like “demand for family planning” since the underlying surveys ask women about pregnancy timing and not their contraceptive desires (Speizer, Bremner, and Farid 2022). It typically includes all women who are using contraception, and, among women not using contraception, women who state that they do not wish to have children within the next two years, and women who were pregnant or postpartum and did not want to become pregnant at the time of their most recent pregnancy (Bradley et al. 2012). We agree with shifting to language about women's desires to replace the old jargon, but adopting a new phrase does not address flaws in the construct: some women using contraception report wanting a child in the next nine months (Canning and Karra 2023) and the construct excludes some women who want to avoid pregnancy for less than two years.
Undesired pregnancy/birth	Unplanned or unintended pregnancy/birth	A pregnancy that occurred when a woman did not want to become pregnant with a/another child. An undesired birth refers to a birth from an undesired pregnancy, though some births may have been desired after a woman learned she was pregnant.
Modern contraception		We consider the following methods to be forms of modern contraception: male and female sterilization, intra‐uterine devices, implants, injectables, oral contraceptive pills, male and female condoms, vaginal barrier methods (including the diaphragm, cervical cap and spermicidal foam, jelly, cream, and sponge), lactational amenorrhea method, emergency contraception, and other modern methods not reported separately (e.g. the contraceptive patch or vaginal ring) (United Nations 2024a).
Contraceptive prevalence rate		The proportion of women of reproductive age (age 15–49 years) who are using any kind of contraception. This metric can be specified to only include modern or traditional contraceptive methods. Contraceptive prevalence of any method was used to monitor progress towards achieving Target 5.B of the Millennium Development Goals (MDG)—achieve, by 2015, universal access to reproductive health (United Nations 2007). The Sustainable Development Goals (SDG) shifted to measuring progress towards the use of modern methods of contraception.
Women who want to avoid pregnancy and are not using any contraception	Unmet need for family planning	Measures the proportion of women of reproductive age who want to avoid pregnancy who are not using any form of contraception. It also includes women who are pregnant or postpartum and who stated that they did not want to become pregnant at the time of their most recent pregnancy. This indicator was used to monitor progress towards achieving MDG Target 5.B.
Modern contraceptive use among women who want to avoid pregnancy	Demand for family planning satisfied by modern methods	Measures the proportion of women of reproductive age who want to avoid pregnancy who are using a modern contraceptive method. The demand for family planning satisfied by modern methods is currently included as Indicator 3.7.1 of the Sustainable Development Goals and is used to monitor progress towards achieving target 3.7—ensure universal access to sexual and reproductive health‐care services, including family planning, information, and education, and the integration of reproductive health into national strategies and programs (United Nations 2017). It is also used as the family planning tracer indicator for SDG 3.8.1—Universal Health Coverage service coverage index.

## DATA AND METHOD

Although our analysis reports undesired *birth* rates, estimating the impact of contraception and abortion on trends in undesired births also requires estimating *pregnancy* rates. However, the available data refer not to pregnancies but to births and abortions. Since fecundity, sexual activity, and contraceptive use shape pregnancy, while pregnancy rates and the percentage of pregnancies aborted affect birth and abortion rates, the Guttmacher Institute and WHO developed a Bayesian hierarchical time series model that concurrently estimates pregnancies by desire and outcome, with global family planning indicators as predictors, that accounts for kind, quality, and availability of data (Bearak et al. 2019, 2020, 2022).

For this study, we updated the model to produce new estimates. Although antecedent literature describes this statistical model, we provide a general overview of the data and model as a foundation to explain how we use this model to produce counterfactual in addition to empirical estimates. The data search and classification process and the data models, which describe the statistical relationships between each datum and its corresponding model‐based estimate remain unchanged (Bearak et al. 2019, 2020).

### Data

Data inputs into the model on abortion, births, and contraceptive use come from several sources, which we systematically assess and classify based on variable amounts of uncertainty and bias. Data inputs on abortion are classified either as *points* when they are from complete official statistics or reliable published studies, and most other sources are treated as *minima* in the model. Official statistics on abortion are of variable quality, particularly in restrictive abortion contexts, which is why some are considered incomplete and not treated as point estimates. In the case of births, data may systematically exclude the individuals most likely to experience undesired pregnancy; for example, a survey that excludes unmarried women likely underestimates the actual proportion of births from undesired pregnancies. We do not adjust these data and instead describe its characteristics to the model: for example, if a datum is a point estimate, then the actual value is equally likely above or below the reported value, but if a datum is a minimum then the actual value is likely no smaller than the reported value.

Overall birth rates come from the World Population Prospects (United Nations Department of Economic and Social Affairs, Population Division 2024b), and data on the percentage of pregnancies/births desired come from surveys. For most low‐ and middle‐income countries, these surveys were the Demographics & Health Surveys (DHS), Multiple Indicator Cluster Surveys, and Reproductive Health Surveys. We also processed the National Surveys of Family Growth (NSFG) and the Encuesta Nacional de la Dinámica Demográfica for the United States and Mexico. We searched the literature for additional data for these and all other countries. Altogether, we calculated from survey data or retrieved from published studies 682 observations of the percentage of pregnancies/births undesired from 142 countries. Of these, 486 observations from 127 countries were points, whereas the remainder provided information on either the minimum or both the minimum and maximum percentage undesired. Before the first DHS, none of the large national survey programs, except the NSFG in the United States, collected data on undesired births; instead, the antecedent of the DHS, the World Fertility Surveys, asked women whether, when they became pregnant, they wanted to *stop* childbearing. We entered percentages computed from these surveys as minima since they coded births to women who wanted to postpone or space childbearing as wanted births. Among published studies, some data also sampled pregnancies instead of births. Since women are less likely to report on pregnancies ending in abortion than those ending in birth, we treated these as minimum estimates of the proportion of pregnancies undesired. A few studies used the London Measure of Unplanned Pregnancy, and, in such instances, the proportion of undesired births falls between the two values called “unwanted” and “planned” that were estimated using that measure; to reflect this, we input those data as range estimates.

Abortion data generally come from official statistics and published studies. Abortion rates calculated from surveys of women underestimate the actual rate (Lindberg et al. 2020; Jones & Forrest 1992; Rossier 2003). As a result, we generally input such data as minima, except for countries in Eastern Europe, Central Europe, and Central Asia, where we modeled a bias term. Countries with robust health systems in which legal abortion is broadly available generally publish reliable abortion statistics. Still, there are exceptions (Popinchalk, Beavin, and Bearak 2021). Altogether, we obtained 2886 observations of abortion incidence from 108 countries. Of these, 1008 observations from 36 countries were official statistics that we classified as having complete abortion counts. Another 50 observations of abortion incidence from 25 countries were from published studies that produced estimates we treated as point estimates, almost always using the Abortion Incidence Complication Methodology developed by Susheela Singh (Singh et al. 2010). Another 27 observations came from surveys of women conducted in 11 countries in Eastern Europe, Central Europe, and Central Asia.

Across all types of data, we obtained 3568 observations of abortion incidence or the proportion of pregnancies/births undesired from 166 countries. Half of these countries (84 countries) had data on *both* types of pregnancy outcomes—abortion, on the one hand, and undesired births, on the other hand. Concurrently estimating *both* pregnancy outcomes—births and abortions—allows us to make maximal use of these data.

### Statistical Model

As predictors in our model, we used the latest set of internationally comparable family planning indicators (United Nations Department of Economic and Social Affairs, Population Division 2024a). These indicators categorize women according to their marital/union status, contraceptive use of modern or traditional methods, and whether they want to avoid pregnancy among those not using contraception. Among unmarried/not‐in‐union women, we use three groups: those not at risk of undesired pregnancy, those at risk of undesired pregnancy but not using a modern method of contraception, and those using modern contraception. Among married/in‐union women, we further distinguish women using traditional contraception from those not using any method, obtaining four groups. We estimate the incidence of pregnancy, Ω, as the sum of all pregnancies among the seven population groups, subscripted w, for each of 186 countries, subscripted c, and ten five‐year periods, subscripted t:

$$\Omega_{ct}=\sum_{w}\omega_{ctw}\times W_{ctw}.$$

In this equation, ω equals a group‐specific pregnancy rate and w is the number of women in that population group. The number of pregnancies in a population group is simply the product of the group‐specific pregnancy rate, $\omega_{ctw}$, and the size of that group, $W_{ctw}$.

The underlying data refer to abortions and births. Therefore, to fit these parameters to the data, we also estimate proportions ending in abortion, denoted α. For the number of abortions, Ψ, we simply multiply out:

$$\Psi_{ct}=\sum_{w}\omega_{ctw}\times W_{ctw}\times \alpha_{ctw}.$$

We followed an approach derived from life tables of pregnancy loss by gestational age to relate the number of pregnancies, $\Omega_{ct}$, and the number of abortions, $\Psi_{ct}$, to the corresponding number of births, $\Theta_{ct}$, with one fetal loss for every five births and another for every 10 abortions: $\Theta_{ct}=\frac{\Omega_{ct}-1.1\Psi_{ct}}{1.2}$ (Bongaarts and Potter 1983; Bongaarts 2015; Dellicour et al. 2016). Using this approach, $\Theta_{ct}$ we calculated group‐specific birth rates, $\theta_{ctw}$:

$$\theta_{ctw}=\frac{\omega_{ctw}-\omega_{ctw}\times \alpha_{ctw}\times 1.1}{1.2}.$$

With these group‐specific rates, we could, in turn, calculate group‐specific counts, $\Theta_{ctw}$:

$$\Theta_{ctw}=\theta_{ctw}\times W_{ctw}.$$

To account for differences between countries and changes over time in the contraceptive method mix, we replaced the family planning indicator for the number of married/in‐union women using modern contraceptives with a number equal to its product with a hypothetical failure rate. To calculate these, we took the cross‐product of method mix data from the *World Contraceptive Use* dataset (United Nations Department of Economic and Social Affairs, Population Division) and method‐specific failure rates from the latest edition of *Contraceptive Technology* (Hatcher et al. 2023). We used a hierarchical model that fit these data to a logistic curve to interpolate between years and calculate scores for all countries and years (Sedgh et al. 2016).

We allow the group‐specific pregnancy rates to vary between countries and five‐year periods. However, we made simplifying assumptions to model the proportions of pregnancies ending in abortion $\alpha_{ctw}$ in light of limited reliable data on the characteristics of women obtaining abortions. Specifically, we modeled these percentages by marital/union status, assumed equal time trends in these percentages for both groups, and fixed these values at zero for desired pregnancies.

To relate the modeled parameters $\alpha_{ctw}$ and $\omega_{ctw}$ to the proportions by desire (and marital/union status) in underlying survey data on pregnancies/births, our model sums birth $\Theta_{ctw}$ or pregnancy $\Omega_{ctw}$ incidence across groups and divides by the overall incidence to calculate distributions by subgroup.

These parameters allow us to specify a model that uses data for all combinations of countries and time periods c,t on the numbers of women and births from the United Nations and, for a subset of these countries and periods, data on births by desire and/or marital/union status and abortions. We estimate these parameters using a Bayesian hierarchical time series that jointly estimates $\alpha_{ctw}$ and $\omega_{ctw}$. To exchange information between countries and time periods, we group countries into subclusters, and these subclusters, in turn, into clusters (Supporting Information Appendix Table 1). For example, the hierarchical time series for a pregnancy rate $\omega_{ctw}$ follows a random walk with subcluster‐specific drift $\Delta_{w,r_{c},t}^{\omega }$:

$$\omega_{\left\{w,c,t\right\}}∼N\left(\omega_{\left\{w,c,t-1\right\}}+\Delta_{\left\{w,r_{c},t\right\}}^{\omega },\sigma_{w}^{2}\right),$$

where $r_{c}$ indicates subcluster r of country c.

With more data available, we specify a more flexible statistical model than used to produce the previously published pregnancy rates. The published model estimates an average difference across clusters in the pregnancy rates for married women who want to avoid not using contraception and those using a traditional method of contraception. It applies this difference to the country‐specific estimates for all countries within a cluster, with no change in this difference over time. Also, the variance terms used to draw the published model's hierarchical parameters—for example, $\sigma_{w}^{2}$ in the equation above—are no longer constrained so that the terms for between‐country variance could not exceed those for between‐cluster and between‐subcluster variance; this means that the model now allows larger differences between countries within a subcluster than between clusters or subclusters in the sizes of subgroup‐specific pregnancy rates and proportions of undesired pregnancies ending in abortion.

### Counterfactuals

To produce a counterfactual estimate of the conditional undesired birth rate, we calculate counterfactuals separately by the seven population groups, sum these, and divide by the empirical number of women who wanted to avoid pregnancy.

To calculate counterfactual undesired birth incidence holding constant contraceptive use and method mix, $\Theta_{ct}^{*}$, we multiply the model‐estimated rates for each of the seven population groups counterfactual group sizes $W_{ctw}^{*(t_{0})}$:

$$\Theta_{ct}^{*\left(t_{0}\right)}=\sum_{w\in w^{undesired}}\theta_{ctw}\times W_{ctw}^{*\left(t_{0}\right)}.$$

To calculate the counterfactual group sizes $W_{ctw}^{*(t_{0})}$, it is not enough to use the group sizes from the alternative time period of interest $W_{ct_{0}w}$. This is because the proportion of women wanting to avoid pregnancy changes over time. We need to hold constant not contraceptive use but *contraceptive use relative to the number of women trying to postpone, space, or stop childbearing*. Hence, for a comparison period $t_{0}$, we calculate

$$W_{ctw}^{*\left(t_{0}\right)}=\frac{W_{ct_{0}w}}{\sum_{w\in w^{undesired}}W_{ct_{0}w}}\times \sum_{w\in w^{undesired}}W_{ctw}.$$

For counterfactual estimates of undesired births holding constant the proportions of pregnancies ending in abortion, we subtract from the empirical undesired pregnancy estimates $\Omega_{ctw}^{undesired}$, a counterfactual abortion incidence estimate. To do this, we first calculate the counterfactual abortion incidence, $\Psi_{ctw}^{@(t_{0})}$ before calculating the corresponding number of births $\Theta_{ctw}^{@(t_{0})}$ for each population group:

$$\Psi_{ctw}^{@\left(t_{0}\right)}=\sum_{w\in w}W_{ctw}\times \omega_{ctw}\times \alpha_{ct_{0}w},$$

$$\Theta_{ctw}^{@\left(t_{0}\right)}=\frac{\Omega_{ctw}^{\mathrm{undesired}}-\left(1.1\times \Psi_{ctw}^{@\left(t_{0}\right)}\right)}{1.2}.$$

### Reported Estimates

We used a Markov Change Monte Carlo algorithm, implemented using JAGS 4.3.2 (Plummer 2023), to generate samples of the posterior distributions of all model parameters, and we carried out our analysis using R 4.4.3 (R Core Team 2025). We computed point estimates and uncertainty intervals by taking medians and the 2.5th and 97.5th percentiles from posterior distributions. This means that when an uncertainty interval excludes zero, 5 percent of the possibility values fall outside the interval, with an equal proportion on either side, and, when the estimates make comparisons such as between two periods, there is at least a 97.5 percent chance that the true change in the value was in the median estimated direction. The uncertainty in the estimates relates to the number of pregnancies by outcome, as the family planning indicators are treated as fixed.

We report annual rates for each five‐year period from 1975–1979 to 2020–2024. To distinguish these from the counterfactuals, we refer to these as empirical estimates. In our main counterfactual analysis, we set $t_{0}$ to 1994‐1994, because the ICPD occurred in this period, and because of increased data scarcity in the 1970s and early 1980s, and include results with $t_{0}$ set to 1975–1979 in the Supporting Information Appendix.

In our text, regions refer to SDG regions, and subregions refer to a country's lowest level regional grouping (subregion or intermediate region) in the UNSD M49 standard (United Nations 2021). To produce regional and subregional rates, we sum the undesired births among all countries in a region or subregion and divide by the sum across countries of women who wanted to avoid pregnancy. All figures for inclusion with the main body text report estimates for SDG regions. Supporting Information figures (labeled S1, S2, S5, and S6), correspond to these figures but show subregional estimates. Finally, we included an appendix with tables that report for all regions and subregions: median estimates with 95 percent uncertainty intervals, posterior probabilities of decrease, probabilities that counterfactual rates would have been higher than empirical rates, and probabilities that counterfactual declines would have been less than empirical declines, in the conditional undesired birth rate.

## RESULTS

Worldwide, about 36 million (95 percent uncertainty interval [UI]: 32–40) million undesired births took place each year on average in 2020–2024 (Supporting Information Appendix Table 2). This corresponds to an annual rate of 32 (29–36) undesired births per thousand women who wanted to avoid pregnancy (Table 2 and Supporting Information Appendix Table A3).

**TABLE 2:** Summary of global and regional results

	Conditional undesired birth rate		Ratio of abortions to undesired births		Percentage of women wanting to avoid pregnancy using modern contraception		Total fertility rate (lifetime births per woman)	
	1990–1994	2020–2024	1990–1994	2020–2024	1990–1994	2020–2024	1990	2024
World	61	32	1.2	2.1	69%	77%	3.3	2.2
Central and Southern Asia	85	21	0.9	4.2	56%	74%	4.3	2.2
Eastern and Southeastern Asia	43	16	1.7	4.3	83%	86%	2.6	1.3
Europe, North America, Australia, New Zealand	22	20	3.1	1.2	71%	81%	1.8	1.4
Latin America and the Caribbean	91	41	0.7	1.3	69%	83%	3.2	1.7
Northern Africa and Western Asia	116	51	1.3	2.0	48%	64%	4.4	2.7
Oceania	131	74	0.4	0.6	39%	51%	4.7	2.9
Sub‐Saharan Africa	174	103	0.4	0.8	26%	56%	6.3	4.2

NOTE: For uncertainty intervals, see Supporting Information Appendix Table A3 for conditional undesired birth rates, Supporting Information Appendix Table A5 for the ratio of abortions to undesired births, and Supporting Information Appendix Table 4b for the percentage of women wanting to avoid pregnancy using modern contraception.

Conditional rates varied substantially across world regions and ranged from 16 (UI: 9–24) undesired births per thousand women who wanted to avoid pregnancy in Eastern and Southeastern Asia, to 103 (93–113) in sub‐Saharan Africa, annually in 2020–2024. The disparities we find across regions do not simply reflect differences between high‐income and other regions. We found similar conditional undesired birth rates in Europe, Northern America, Australia, and New Zealand and Central and Southern Asia, where annual rates were 20–21 (17–24; 15–28), and the smallest rate of 16 (9–24) in Eastern and Southeastern Asia, annually per thousand women who wanted to avoid pregnancy in 2020–2024. However, the greatest rate by far is in the region with the most low‐income countries, sub‐Saharan Africa.

**FIGURE 1:**
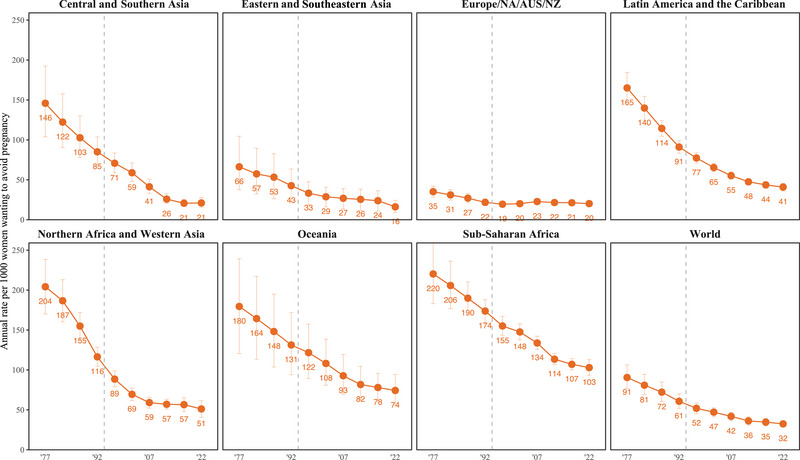
Annual rate of undesired births per 1000 women who want to avoid pregnancy (the conditional undesired birth rate) by the SDG region and globally, 1975–1979 to 2020–2024. NOTES: Solid vertical lines show 95 percent uncertainty intervals. The gray‐dashed vertical line shows 1994, the year of the International Conference on Population and Development.

As substantial as differences across regions were in 2020–2024, more substantial differences were evident in earlier years. Around the time the ICPD Programme of Action was adopted, rates ranged from 22 (UI: 18–26) in Europe, Northern America, Australia, and New Zealand to 174 (160–188) in sub‐Saharan Africa, annually in 1990–1994. In the earliest period for which we produced estimates, 1975–1979, annual rates ranged from 35 (28–42) to 220 (183–259) undesired births per thousand women who wanted to avoid pregnancy in these subregions (Figure 1 and Supporting Information Appendix Table 3).

Disparities between regions in the rate of undesired births per thousand women who wanted to avoid pregnancy declined since before the ICPD because declines were greatest, measured on absolute as well as relative scales, in the regions with the highest rates. We found the largest absolute decline in Northern Africa and Western Asia, from 204 (UI: 170–238) to 51 (41–61). The next‐largest declines happened in Latin America and the Caribbean, where the rate fell from 165 (145–185) to 41 (36–46), and in Central and Southern Asia, where it fell from 146 (104–193) to 21 (15–28) undesired births per thousand women who wanted to avoid pregnancy. Across all regions, we found the smallest decline in Europe, Northern America, Australia, and New Zealand, where the rate fell from 35 (28–42) to 20 (17–24). On a relative scale, we found substantial but much smaller variation between regions in these trends. Relative decreases ranged from 43 percent (27–54 percent) in the region that includes Europe to 86 percent (78–90 percent) in Central and Southern Asia.

While declines were generally greater among the regions with higher rates, there were differences in progress among these regions since the 1970s. During the earliest period we analyze, 1975–1979, sub‐Saharan Africa and Northern Africa and Western Asia had much more similar conditional undesired birth rates. Since then, however, we found much less progress in sub‐Saharan Africa. In Northern Africa and Western Asia, the rate declined by 75 percent (UI: 68–81 percent). By contrast, in sub‐Saharan Africa, the rate declined 53 percent (42–61 percent), from 220 (183–259) undesired births annually per thousand women who wanted to avoid pregnancy in 1975–1979 to 103 (93–113) in 2020–2024. As a result, about twice as many undesired births take place each year in sub‐Saharan Africa than in Northern Africa and Western Asia, annually per thousand women who want to avoid pregnancy in 2020–2024. However, almost all of the decreases in Northern Africa and Western Asia occurred before 2005–2009, when the rate reached 59 (52–66). In sub‐Saharan Africa, by contrast, rates continue to decline at the same relative pace.

### Slowing Paces of Decline in Conditional Undesired Birth Rates

**FIGURE 2:**
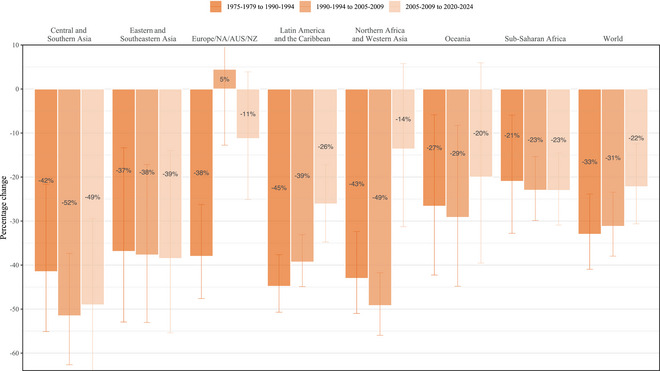
Relative change (percent) in the annual rate of undesired births per 1000 women who want to avoid pregnancy (the conditional undesired birth rate) by the SDG region and globally, 1975–1979 to 2020–2024 NOTES: Vertical lines show 95 percent uncertainty intervals. The upper limits of some intervals extend beyond the *y*‐axis limits of this figure, and the Supporting Information Appendix tables report these and other values.

We found slowing paces of decline in conditional undesired birth rates in several middle‐income regions beginning in the early 2000s. In Latin America and the Caribbean, Northern Africa and Western Asia, and Oceania, the differences between adjacent 15‐year periods on a relative scale became much shallower. The most pronounced slowdown occurred in Northern Africa and Western Asia. In this region, the conditional undesired birth rate fell by 49 percent (UI: 42–56 percent) between 1990–1994 and 2005–2009 (Figure 2). Thereafter, we found a three‐and‐a‐half‐fold slowdown in progress and just a 91 percent probability that any decline occurred in the conditional undesired birth rate after 2005–2009 (Supporting Information Appendix Table 3). In Latin America and the Caribbean, by contrast, we saw much less stagnation. In this region, the conditional undesired birth rate declined by 26 percent (17–35 percent) between 2005–2009 and 2020–2024. In Oceania, meanwhile, although we find less evidence of change after 2005–2009 than in preceding periods, the uncertainty interval for the rate of change substantially overlaps with the amount of change estimated over preceding periods.

In addition to these regions, declines in conditional undesired birth rates stagnated in Europe, Northern America, Australia, and New Zealand. There, however, progress began to stagnate much earlier. Its conditional undesired birth rate decreased by 38 percent (26–48) from 1975–1979 to 1990–1994, with an increase more likely than a decrease between 1990–1994 and 2005–2009.

Comparing the proportionate decreases from three 15‐year intervals in our analysis, 1975–1979 to 1990–1994, 1990–1994 to 2005–2009, and 2005–2009 to 2020–2024, we find that, among regions where the evidence does not point to plateauing gains, our estimates are overall more consistent with sustained progress in contrast to accelerating progress after the ICPD. Evidence of an increase in the proportionate trends before and after ICPD in sub‐Saharan Africa and Eastern and Southeastern Asia was slight, with approximately the same proportionate gains. However, we saw some evidence of an increase in the rate of progress in Central and Southern Asia. In this region, the conditional undesired birth rate declined 52 percent (UI: 37–63 percent) from 1990–1994 to 2005–2009, having previously fallen by 42 percent (22–55 percent) from 1975–1979 to 1990–1994.

More variation exists comparing subregional trends than regional trends, such that among the subregions of sub‐Saharan, we find more trend variation between subregions within this region than we do across regions worldwide. Dramatic declines in the conditional undesired birth rate occurred in most African subregions (Supporting Information Figure S1 and Appendix Table 3). Yet, in Middle Africa, the subregion of sub‐Saharan Africa which had the lowest conditional undesired birth rate in 1975–1979, the stagnating progress resulted in it having the highest rate by far among African subregions, south or north of the Sahara, in 2020–2024: 163 (UI: 138–191) undesired births annually per thousand women who wanted to avoid pregnancy. There, we estimated a mere 74 percent chance that any decline occurred over the analysis period. We also find evidence of stagnating progress in Southern Africa, where, over the last 15 years, we found a 91 percent chance that the conditional undesired birth rate decreased; correspondingly, the uncertainty interval for the decrease (10 percent [–5 to 24 percent]) included zero.

In Central and Southern Asia, the overall decline in the conditional undesired birth rate heavily reflects trends in Southern Asia. In this subregion, the rate fell from 152 (UI: 107–201) undesired births annually per thousand women who wanted to avoid pregnancy in 1975–1979 to 21 (16–28) in 2020–2024. In Central Asia, by contrast, we found a rate of 35 (16–64) and 13 (6–22) in these same periods. This still represents a substantial, 63 percent (7–85 percent) decline. Earlier on, however, in 2005–2009, 10 (7–14) undesired births took place annually per thousand women who wanted to avoid pregnancy in Central Asia, meaning the direction of the trend may have possibly reversed. By contrast, in Southern Asia, the rate fell by half again (50 percent [30–65 percent]) after 2005–2009.

Finally, in Europe, Northern America, Australia, and New Zealand, we discussed evidence of dramatic relative declines before the onset of stagnation in the 1990s. In Northern America, however, we find little evidence of change throughout the analysis period, with rates increasing and decreasing marginally across periods.

Compared to other regions, we found less of a contrast in the relative trends of the subregions within Latin America and the Caribbean and Northern Africa and Western Asia.

### Trends in Family Planning Indicators

Co‐occurring with trends in undesired births over the past century, all regions saw changes and increases in the proportion of reproductive‐age women who want to avoid pregnancy—meaning they were married or had sex in the past 30 days, fecund, and stated that they wanted to postpone, space, or stop childbearing, or were using a method of contraception.

Worldwide, the percentage of reproductive‐aged women who want to avoid pregnancy increased from 48 percent (UI: 49–52 percent) in 1975–1979 to 57 percent (55–59 percent) in 2020–2024 (Supporting Information Appendix Table 4a). The largest change occurred in Latin America and the Caribbean, where 66 percent (63–69 percent) of women wanted to avoid pregnancy in 2020–2024, an increase from 44 percent (40–48) of women in 1975–1979. The smallest change occurred in Europe, Northern America, Australia, and New Zealand, where today, 65 percent (62–70 percent) of women want to avoid becoming pregnant.

Declines in the proportion of women who want to avoid pregnancy not using *any* method of contraception, modern or traditional, co‐occurred with increases in the proportion of women who wanted to avoid pregnancy. The largest change in both indicators occurred in sub‐Saharan Africa. There, the proportion not using any contraceptive decreased from 73 percent in 1975–1979 to 36 percent in 2020–2024 (Supporting Information Appendix Table 4d). *Modern* contraceptive use in sub‐Saharan Africa meanwhile rose fivefold among women who want to avoid pregnancy, from 11 percent (UI: 7–18 percent) of women who wanted to avoid pregnancy in 1975–1979 to 56 percent (54–58 percent) in 2020–2024 (Supporting Information Appendix Table 4b).

**FIGURE 3:**
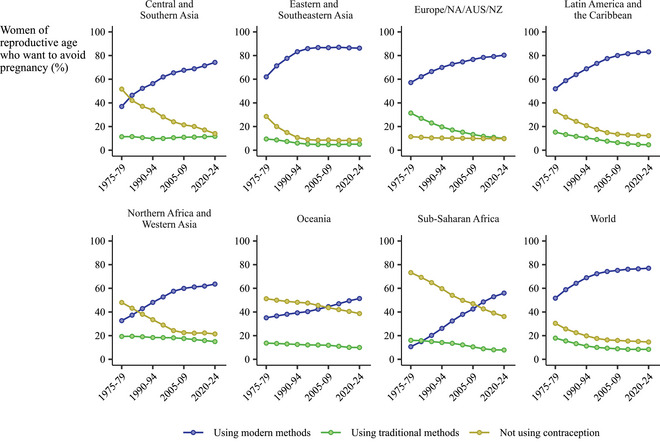
Contraceptive use by method type and contraceptive nonuse among women who want to avoid pregnancy by the SDG region and globally, 1975–1979 to 2020–2024

In all regions except Europe, Northern America, Australia, and New Zealand, the use of some form of contraception among women who want to avoid pregnancy increased from the levels observed in 1975–1979 (Figure 3). For modern contraceptives specifically, we saw increased use in this region. Eastern and Southern Europe also saw shifts away from the use of traditional methods in favor of modern contraceptives throughout our analysis period. We also saw shifts from traditional to modern methods in Western and Northern Europe up through the 1980s.

Worldwide on average among women who wanted to avoid pregnancy, the share who were not using modern contraception decreased by 36 percent in the first third of the periods we analyzed, from 48 percent (UI: 45–52 percent) of women who wanted to avoid pregnancy in 1975–1979 to 31 percent (29–34 percent) in 1990–1994. The global average decreased another 20 percent in the middle third to 25 percent (23–27 percent) in 2005–2009. Then, it decreased 7 percent in the remaining interval to 23 percent (21–25 percent) in 2020–2024.

**FIGURE 4:**
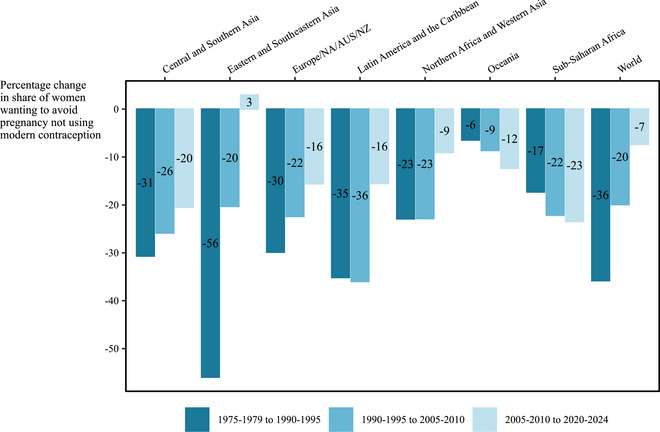
Relative change (percent) among women who want to avoid pregnancy in the percentage not using modern contraception by the SDG region and globally, 1975–1979 to 2020–2024

We observed a slowing proportionate rate of change in the share of women who were not not using modern contraception, among those who wanted to avoid pregnancy, in most regions (Figure 4). The most pronounced plateauing occurred in Eastern and Southeastern Asia, where modern contraceptive use peaked in the middle interval. Sustainment or acceleration in the proportionate rate of change occurred only in sub‐Saharan Africa and Oceania.

Slowing gains in contraceptive use among women who want to avoid pregnancy in Northern Africa and Western Asia and Latin America and the Caribbean after 2005–2009 correspond to slowing paces of decline in conditional undesired birth rates. In contrast, in Europe, Northern America, Australia, and New Zealand, declines in conditional undesired birth rates stalled even when modern contraceptive use did not plateau. In other regions, conditional undesired birth rates continue to decline by similar relative amounts as in previous periods, even as relative decreases in contraceptive nonuse slowdown. Thus, we found that contraceptive plateauing occurred more widely than stagnating declines in conditional undesired birth rates.

### Trends in Abortion

**FIGURE 5:**
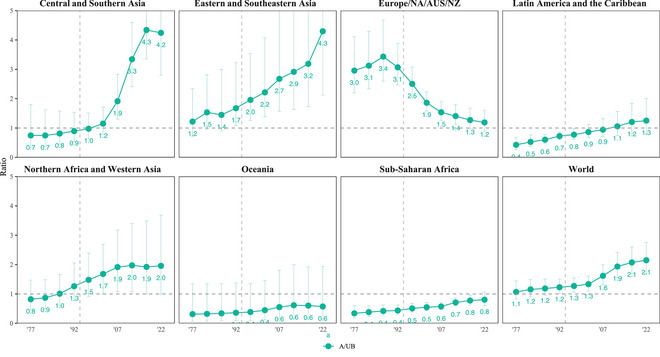
Trends in the ratio between abortions and undesired births by SDG region and globally, 1975–1979 to 2020–2024 NOTES: Solid vertical lines show 95 percent uncertainty intervals. The upper limits of some intervals extend beyond the y‐axis limits of the figure, and the Supporting Information Appendix tables report these and other values. The gray‐dashed vertical line corresponds to 1994, the year of the International Conference on Population and Development.

Changes in the use of abortion after an undesired pregnancy explain why we see trends in undesired births that do not align with trends in contraceptive use among women who want to avoid pregnancy. In Europe, Northern America, Australia, and New Zealand, the use of abortion peaked in 1985–1989, when there were 3.4 as many abortions as undesired births (Figure 5 and Supporting Information Appendix Table 5). Since then, the use of abortion following an undesired pregnancy declined so much in this region that there were only 1.2 (UI: 0.9–1.6) abortions for every undesired birth in 2020–2024. In other words, modern contraceptive use increased, reducing the probability a woman who wanted to avoid pregnancy would nonetheless become pregnant, but among those who became pregnant, they became less likely to want or be able to obtain an abortion than they were in the 1980s. In all other regions, we estimated increased use of abortion, which more than offset slowdowns in contraceptive uptake.

**FIGURE 6:**
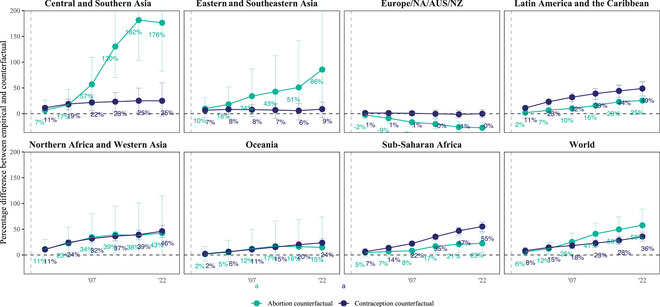
Difference (percent) between empirical and counterfactual estimates of the conditional undesired birth rate by the SDG region and globally, 1990–1994 to 2020–2024 NOTES: The contraception counterfactual calculates a counterfactual conditional undesired birth rate, $\Theta_{ctw}^{*(t_{0})}$, by applying the contraceptive use and method mix among women at risk of undesired pregnancy from 1990 to 1994 to women at risk of undesired pregnancy in each other period. As a result, the rate of undesired pregnancy changes, affecting the rate of undesired births. In contrast, the abortion counterfactual, $\Theta_{ctw}^{@(t_{0})}$ uses the empirical estimates of undesired pregnancy but applies the percentage of undesired pregnancies ending in abortion, by marital/union status, from 1990−1994. The percentage difference for any time period t equals $\frac{\Theta_{t}^{*(1990-1994)}-\Theta_{t}}{.01\times \Theta_{t}}$ or $\frac{\Theta_{t}^{@(1990-1994)}-\Theta_{t}}{.01\times \Theta_{t}}$ for contraception and abortion, respectively. Solid vertical lines show 95 percent uncertainty intervals. The upper limits of some intervals extend beyond the *y*‐axis limits of the figure, and the Supporting Information Appendix tables report these and other values. The gray‐dashed vertical line corresponds to 1994, the year of the International Conference on Population and Development.

Increases in the use of abortion following an undesired pregnancy were most pronounced in Asia. In both Central and Southern Asia and Eastern and Southeastern Asia, the ratio of abortions to undesired births exceeded 4 in 2020–2024. With subregional variation, however, the overall trends in Asia did not well characterize trends in Central Asia. In that subregion, declines in abortion incidence far outpaced declines in the number of undesired births (Supporting Information Figure S5). As a result, the abortion ratio in Central Asia fell from 7.9 (UI: 4.0–18.2) abortions per undesired birth in 1975–1979 to 4.3 (2.2–9.2). Trends in Central Asia were more comparable to trends in Eastern Europe, where the ratio fell from 7.4 (4.7–13.0) abortions per undesired birth in 1975–1979 to 2.2 (1.2–4.2) in 2020–2024. In other Asian subregions, abortion ratios tripled, quadrupled, or sextupled.

Because of the increased use of abortion following an undesired pregnancy in low‐ and middle‐income regions despite heavy restrictions, the abortion ratio in Latin America and the Caribbean now exceeds that found in Europe, Northern America, Australia, and New Zealand. For every undesired birth that occurred in Latin America and the Caribbean in 2020–2024, 1.3 (UI: 0.8–2.0) abortions took place. Comparing subregions, we find an abortion ratio in Western Europe (0.8 [0.6–1.1]) equal to or smaller than that in some African subregions–specifically, Eastern Africa (0.8 [0.6–1.2]), Western Africa 1.1 (0.7–1.7), and Northern Africa (1.2 [0.5–3.3]). Considering substantial uncertainty in these ratios, rates in these more‐ and less highly resourced settings could well be more dissimilar than we estimate. This uncertainty reflects a lack of reliable abortion data in Africa, Latin America, and the Caribbean and Asia, mitigated by several abortion incidence studies conducted with increasing rarity, and limited data on the proportions of births undesired in Western, Northern, and Southern Europe.

### The Impact of Abortion and Contraception on Undesired Births

In the 30 years since ICPD, the global average conditional undesired birth rate declined by 46 percent (UI: 38–53 percent), from 61 (52–70) to 32 (29–36) undesired births per thousand women who wanted to avoid pregnancy (Supporting Information Appendix Table 3). Had contraceptive use not changed after 1990–1994, the conditional undesired birth rate would have only decreased by 27 percent (15–37 percent) to 44 (40–49) undesired births per thousand women who wanted to avoid pregnancy. Had the use of abortion following an undesired pregnancy not changed after 1990–1994, the conditional undesired birth rate would have only decreased by 15 percent (0–28 percent) to 51 (43–62) undesired births per thousand women who wanted to avoid pregnancy. Compared to the empirical trend, thus, had contraceptive use not changed, the empirical decline would have been 41 percent (28–61 percent) smaller, whereas had the use of abortion following an undesired pregnancy not changed, the empirical decline would have been 67 percent (41–100 percent) smaller.

Compared to the level of undesired births in 2020–2024, the global undesired birth rate would have been 58 percent (34–89 percent) greater had it not been for abortion trends or 36 percent (27–44 percent) higher in 2020–2024 had it not been for contraceptive trends (Figure 6 and Supporting Information Appendix Table 6). Put differently, abortion had 1.6 times the impact of contraception on reducing the global undesired birth rate since ICPD.

The impact of abortion on trends in undesired births varied dramatically in magnitude and direction. Had the use of abortion following an undesired pregnancy not changed over time in Europe, Northern America, Australia, and New Zealand, its conditional undesired birth rate would have declined substantially more than it did. Empirically, we found similar rates in 1990–1994 and 2020–2024, when the rate was 20 (UI: 17–24). Counterfactually, had the use of abortion following an undesired pregnancy not changed over this period, its rate would have declined 33 percent (UI: 21–42 percent) from 22 (18–26) to 15 (12–17) to undesired births per thousand women who wanted to avoid pregnancy, instead of staying about the same.

In all other regions, in contrast to Europe, Northern America, Australia, and New Zealand, the use of abortion following an undesired pregnancy helped drive declines in conditional undesired birth rates. Among those other regions, excepting Oceania, whose estimates had more uncertainty, the probability that the counterfactual exceeded the empirical trend ranged from 97.5 percent in Northern Africa and Western Asia to more than 99.9 percent in Central and Southern Asia. 

We found the largest contributions of abortion in Central and Southern Asia and Eastern and Southeastern Asia. There, holding contraceptive use among women who wanted to avoid pregnancy steady from 1990 to 1994 would have had minimal to no impact on trends in the conditional undesired birth rate, while holding constant the use of abortion following an undesired pregnancy would have resulted in a 32 percent (UI: 0–53 percent) decline over time in the rate in Central and Southern Asia to a rate of 58 (40–84), and a 28 percent (‐8–53 percent) decline over time in Eastern and Southeastern Asia, to a rate of 30 (15–55), in 2020–2024. These counterfactual declines in undesired birth rates contrast with much larger empirical declines: since ICPD, the conditional undesired birth rate fell by 75 percent (65–83 percent) in Central and Southern Asia, from 85 (69–104) to 21 (15–28), and by 62 percent (44–74 percent) in Eastern and Southeastern Asia, from 43 (22–63) to 16 (9–24) undesired births per thousand women who wanted to avoid pregnancy. Had the use of abortion following an undesired pregnancy not changed in these regions, thus, the empirical decline in the conditional undesired birth rate after ICPD would have been 58 and 54 percent (31–99 percent; 13–113 percent) smaller, respectively, in these regions.

While contraception had much less impact on the global average trend in the conditional undesired birth rate after ICPD than abortion had, they contributed to regional trends much more equally in other regions than in Asia.

In two SDG regions, sub‐Saharan Africa and Latin America and the Caribbean, we found slightly more evidence that increases in contraceptive use contributed to trends in undesired births than the use of abortion following an undesired pregnancy. However, the uncertainty intervals for the abortion and contraception counterfactuals substantially overlapped. While the strength of the evidence that one factor contributed more than another was thus relatively weak, the evidence that each of these determinants contributed to declines in undesired birth rates was comparatively strong. Empirically in sub‐Saharan Africa, the conditional undesired birth rate fell 41 percent (UI: 33–48 percent) from 174 (160–188) in 1990–1994–103 (93–113) in 2020–2024. In Latin America and the Caribbean, the conditional undesired birth rate fell 55 percent (49–61 percent), from 91 (83–99) in 1990–1994 to 41 (36–46) in 2020–2024. Had the use of abortion following an undesired pregnancy not changed in these regions, the conditional undesired birth rate in 2020–2024 would have been 126 (107–147) undesired births per thousand women who wanted to avoid pregnancy in sub‐Saharan Africa, 23 percent (5–43 percent) higher than its empirical rate, and 51 (42–65) undesired births per thousand women who wanted to avoid pregnancy in Latin America and the Caribbean, 10 percent (0–24 percent) higher than its empirical rate. Had contraceptive use not changed in these regions, the conditional undesired birth rate would have been greater still, declining only to 160 (142–177) undesired births per thousand women who wanted to avoid pregnancy in sub‐Saharan Africa, 55 percent (45–64 percent) higher than its empirical rate, and 61 (52–70) undesired births per thousand women who wanted to avoid pregnancy in Latin America, 49 percent (34–62 percent) higher than its empirical rate, in 2020–2024.

Overall, we found much less variation between regions in the impact of contraception compared to abortion on trends in undesired births. However, the extent of variation in the impact of abortion was largely due to patterns in Asia. Elsewhere, contraception and abortion contributed much more similarly to trends in undesired births.

## DISCUSSION

Our analysis reveals new insight regarding global temporal trends in undesired births, contraceptive use, and abortion between 1975 and 2024. Overall, we do not find that the pace of decline in the conditional undesired birth rate accelerated following the ICPD. Conditional undesired birth rates were decreasing before then. In the following years, global average conditional undesired birth rates continued to decrease, at roughly the same proportionate pace. However, from 2005–2009 to 2020–2024, the pace of decline slowed, as the global average conditional undesired birth rate decreased by 22 percent (12–31), compared to 31–33 percent (23–38; 24–41) in the previous 15‐year intervals.

In the 30 years since the ICPD and the Beijing Declaration, we find that abortion contributed to declines in undesired birth rates in most regions, including those with highly restrictive abortion laws. Over this period, knowledge about and increases in the availability of medication abortion through the use of misoprostol with or without mifepristone contributed to the accessibility and safety of abortion (Ganatra et al. 2017). Initially developed for ulcer treatment, misoprostol has long been on WHO's core list of essential medicines (Gill, Ganatra, and Althabe 2019). Misoprostol's affordability, effectiveness, and availability in restrictive settings likely contributed to increasing proportions of undesired pregnancies ending in abortion in restrictive settings (Bearak et al. 2020; Moseson et al. 2024; Raymond et al. 2019; Starrs et al. 2018).

Whereas worldwide, on average, abortion contributed more than contraception to decreases in undesired birth rates, global averages obscure substantial variation. The use of abortion following an undesired pregnancy has increased dramatically in Southern, Southeastern, and Eastern Asia since the mid‐2000s. In contrast, the heavy dependence on abortion in Eastern Europe and Central Asia up until the 1990s dramatically declined; there, the substitution of contraception for abortion, as a broader range of contraceptive methods became more broadly available, largely explains the observed trend.

The timing of donor shifts in funding for family planning programming away from middle‐income countries, such as Latin America and the Caribbean and Northern Africa and Western Asia, corresponds roughly with when plateaus began there. The United States government was the largest donor for family planning programming. By 2012–2017, the US Agency for International Development (USAID) had “graduated” most countries in Latin America and the Caribbean from family planning assistance, meaning, the agency transitioned countries from aid for family planning when it was believed that governments were ready to take over their programming. In many countries, the discontinuation of donor funding often corresponded with a country transitioning to becoming a middle‐income country and exhibiting achievement according to other metrics, such as total fertility and modern contraceptive prevalence (USAID 2012). In Northern Africa and Western Asia, a similar process unfolded during a similar time with a planned phase‐out of US government support for family planning, broader funding cuts to the region, and a concentration of funding focused on Egypt and Jordan.

These changes in the enabling environment could relate to why, in Latin America and the Caribbean, Oceania, and Northern Africa and Western Asia, conditional undesired birth rates have hovered around 40, 80, and 60, respectively, since the early‐ to mid‐2000s, whereas in high‐income contexts, such as Europe, Northern America, Australia, and New Zealand as well as in some other regions, such as Central and Southern Asia, and Eastern and Southeastern Asia, a plateau occurred as rates approached 20–30 undesired births per thousand women wanting to avoid pregnancy. In Latin America, conditional undesired birth rates would have been higher had it not been for increases in the use of abortion following an undesired pregnancy that continued even as increases in contraceptive use among women wanting to avoid a pregnancy slowed. Continued increases in proportions of undesired pregnancies ending in abortion, which began before the Green Wave movement coalesced and resulted in abortion reforms, kept declines in the undesired birth rate from plateauing to the extent seen in other middle‐income regions.

Undesired birth rates thus appear asymptotic across multiple and highly diverse geographic contexts. Since some people who do not want a pregnancy may choose not to use any method, even a traditional one, and because pregnancies occur to women using contraception, we do not expect ever to have zero undesired pregnancies. And, because individuals may not also wish to use abortion regardless of accessibility, we do expect ever to have zero undesired births. Still, asymptotes could be lower, and population plateaus can mask within‐population inequalities.

This study has several strengths. Our counterfactual approach allowed us to assess the extent to which contraception and abortion contributed to trends. In addition, the vast amount of data we processed made it possible to provide estimates for 15 years before and 10 years after the most recent comparable study (Bearak et al. 2018). In turn, expanding the years analyzed allowed us to examine whether progress found after ICPD had been occurring beforehand or whether trends altered. We also used a new indicator, the conditional undesired birth rate, for our analysis. This indicator better captures trends in alignment between reproductive desires and outcomes by removing the impact of changing fertility desires occurring concurrently. As a result, the differences we find more closely related to women's agency in determining whether and when to bear children than they would in an analysis using the standard metric.

Our analysis also has several limitations. Although we can assess evidence for global progress towards increased alignment between women's reproductive desires and outcomes during earlier years, limitations of the historical data contribute to increased uncertainty in the estimates. We also discussed regional and subregional findings, and the patterns should not be inferred to specific countries. Country‐level trends concerning ICPD's impact, as well as the relative contribution of abortion versus contraception in the post‐ICDP period, can vary widely, as can the amount of uncertainty in the estimates. We see this, for example, through its impact on our estimates of the abortion to undesired birth ratios for Eastern Asia; reliable data on neither abortion nor undesired births exist for its largest country in the post‐ICPD era. Finally, to calculate the counterfactuals that estimate the impacts of abortion and contraception, we hold constant estimable parameters we could fit to the available data, such as the proportions of women at risk of undesired pregnancy using modern contraception by union status, but contraceptive method mix only for women married or in union. With other large‐scale contextual or secular changes, such as those relating to population structure, economic development, or geopolitical instability, that may have occurred which may have altered the impact of ICPD, we stress that these findings are descriptive and not causal.

ICPD galvanized global support for sexual and reproductive health and rights, and we do not doubt that commitments made in response changed norms, improved policies, advanced rights, and saved lives in the decades that followed (Sen 2019). Nevertheless, our results raise concerns about stagnating progress in the alignment between women's population‐average fertility desires and outcomes.

With funding cuts in the early 2000s, USAID and other donors concentrated family planning investments on their priority countries, many of which were in sub‐Saharan Africa (USAID 2012). There, declines in the conditional undesired birth rate continued relatively steadily. Progress slowed in Latin America and would have slowed more had it not been for abortion. Political and economic pressures on donors may affect funding in future years, and resource constraints will likely raise questions about targeting investments.

Our research examines global, regional, and subregional trends in conditional undesired birth rates. Our findings highlight the contributions abortion and contraception had in enabling women to exercise reproductive agency. This and slowing progress related to contraceptive use in regions experiencing disinvestment suggests that progress depends on both, and accelerating progress likely requires more significant investment in these essential sexual and reproductive healthcare services. While our study addresses undesired births, concerns about below‐replacement fertility affect the politics around reproductive rights in some places, as others remain concerned about population growth. More research could address trends in the proportion of the population trying to become pregnant and the corresponding conditional desired birth rates. Future work should also examine the individual, neighborhood, regional, and national‐level social, economic, policy, and regulatory factors contributing to misalignment between reproductive desires and outcomes.

## CONFLICTS OF INTEREST STATEMENT

We declare no conflicts of interests.

## Supporting information



Supporting information

Supporting information
