# Supplementary material for: Undesired Births, Contraception, and Abortion Before and After the Cairo Consensus: Trends in Conditional Undesired Birth Rates and the Impact of Contraception and Abortion
Source: Stud Fam Plann. 2025 May 20;56(2):274–300. doi: 10.1111/sifp.70014 (PMC12205728; doi:10.1111/sifp.70014)
Supplement: Supplementary file 1 — Supporting information [file SIFP-56-274-s002.docx]

APPENDIX FIGURE A1. Proportion of women of reproductive age (15-49 years) who want to avoid pregnancy by SDG region and globally, 1975-1979 to 2020-2024

**
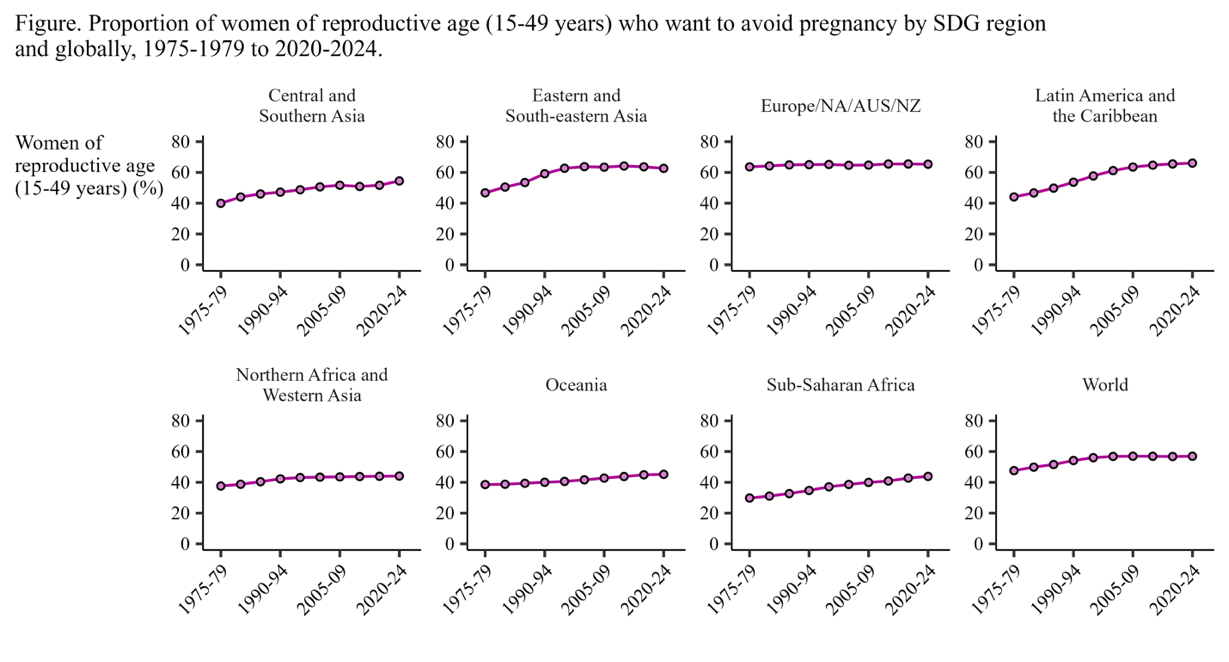
**

APPENDIX FIGURE A2. Empirical and counterfactual estimates of the change (%) in the conditional undesired birth rate from 1990-1994 to 2020-2024 by SDG region and globally

Vertical lines show 95% uncertainty intervals. Upper limits of some intervals extend beyond the y-axis limits of this figure, and the appendix tables report these and other values.

APPENDIX FIGURE A3. Difference (%) between empirical and counterfactual estimates of the conditional undesired birth rate in SDG regions and globally, 1975-1979 to 2020-2024

Solid vertical lines show 95% uncertainty intervals. Upper limits of some intervals extend beyond the y-axis limits of the figure, and the appendix tables report these and other values. Grey dashed vertical line corresponds to 1994, the year of the International Conference on Population and Development.

The contraception counterfactual calculates a counterfactual conditional undesired birth rate, $\Theta_{ctw}^{*\left( t_{0} \right)},$ by applying the contraceptive use and method mix among women at risk of undesired pregnancy from 1975-1979 to women at risk of undesired pregnancy in each other period. As a result, the rate of undesired pregnancy changes, which in turn affects the rate of undesired births. In contrast, abortion counterfactual, $\Theta_{ctw}^{@\left( t_{0} \right)}$, uses the empirical estimates of undesired pregnancy but applies the percentage of undesired pregnancies ending in abortion, by marital/union status, from 1990−1994. The percentage difference for any time period $t$ equals: $\frac{\Theta_{t}^{*(1975-1979)}-\Theta_{t}}{.01 \times\Theta_{t}}$ or $\frac{\Theta_{t}^{@(1975-1979)}-\Theta_{t}}{.01 \times\Theta_{t}}$ for contraception and abortion, respectively.

APPENDIX FIGURE A4. Empirical and counterfactual estimates of the change (%) in the conditional undesired birth rate from 1975-1979 to 2020-2024 by SDG region and globally

Vertical lines show 95% uncertainty intervals. Upper limits of some intervals extend beyond the y-axis limits of this figure, and the appendix tables report these and other values.

SUPPLEMENTAL FIGURE S1. Annual rate of undesired births per 1000 women who want to avoid pregnancy (the conditional undesired birth rate) by M49 region and globally, 1975-1979 to 2020-2024

Solid vertical lines show 95% uncertainty intervals. Grey dashed vertical line shows 1994, the year of the International Conference on Population and Development.

SUPPLEMENTAL FIGURE S2. Relative change (%) in the annual rate of undesired births per 1000 women who want to avoid pregnancy (the conditional undesired birth rate) by M49 region and globally, 1975-1979 to 2020-2024

Solid vertical lines show 95% uncertainty intervals. Grey dashed vertical line shows 1994, the year of the International Conference on Population and Development.

*The next Supplemental Figure after S2 is S5.*

*For estimates by M49 subregion corresponding to results by SDG region in Figures 3 and 4, see Appendix Tables 3a–3d.*

SUPPLEMENTAL FIGURE S5. Trends in the ratio between abortions and undesired births by M49 region and globally, 1975-1979 to 2020-2024

Solid vertical lines show 95% uncertainty intervals. Upper limits of some intervals extend beyond the y-axis limits of this figure, and the appendix tables report these and other values. Grey dashed vertical line shows 1994, the year of the International Conference on Population and Development.

SUPPLEMENTAL FIGURE S6. Difference (%) between empirical and counterfactual estimates of the conditional undesired birth rate by M49 region and globally, 1990-1994 to 2020-2024

Solid vertical lines show 95% uncertainty intervals. Upper limits of some intervals extend beyond the y-axis limits of this figure, and the appendix tables report these and other values. Grey dashed vertical line shows 1994, the year of the International Conference on Population and Development.
